# The Role of Lifestyle, Eating Habits and Social Environment in the Prevention and Treatment of Type 2 Diabetes and Hypertension

**DOI:** 10.3390/nu13051460

**Published:** 2021-04-25

**Authors:** Kalliopi Karatzi, Yannis Manios

**Affiliations:** 1 Department of Nutrition and Dietetics, School of Health Science and Education, Harokopio University, 17671 Athens, Greece; pkaratzi@hua.gr; 2 Institute of Agri-Food and Life Sciences, Hellenic Mediterranean University Research Centre, 71410 Heraklion, Greece

Type 2 diabetes (T2DM) and hypertension are major health problems, with an undisputed growth burden in the past decades. When uncontrolled, both conditions have many consequences regarding health, wellbeing and health care costs. The basis for action to tackle T2DM and hypertension lies in raising awareness regarding the need for early prevention, timely diagnosis and effective treatment. Yet, the lack of supportive environments for healthy lifestyles, and the lack of access to quality health care means, makes design of public health initiatives imperative to promote sustainable lifestyle changes for all.

Although there are several options for drug intervention, lifestyle changes are still the cornerstone of both prevention and treatment. Several nutrients, food items and dietary and lifestyle patterns have proven effective in recent studies, and several mechanisms have been described to support the protective roles of such interventions on both T2DM and hypertension. However, regarding a holistic approach for the prevention and management of both health conditions, we are still at the beginning of discovering the most cost-effective initiatives for promoting sustainable lifestyle changes and ensuring equity and access to healthcare services for all. The special issue entitled *Nutrition and Lifestyle for the Prevention of Type 2 Diabetes and Hypertension*, aimed at gathering novel scientific knowledge regarding prevention or treatment of these diseases through modification of lifestyle behaviors and highlights the environmental and social factors that act either as facilitators or constraints.

Regarding T2DM, several meta-analyses, reviews, observational and intervention studies have highlighted the dietary and lifestyle modifications related to reduced risk. The review by Altobelli et al. [1], highlights the high prevalence of T2DM and its main risk factors in Europe with large variability between European countries and between genders. In the umbrella review of Toi et al. [2], results from 66 reviews and meta-analyses showed that adherence to a healthy dietary pattern like the Mediterranean or the DASH dietary pattern, as well as high consumption of whole grains, low-fat dairy products, yogurt, olive oil, chocolate, fiber and flavonoids, significantly reduced the risk of T2DM. Regarding dietary protein and risk of T2DM, the systematic review and meta-analysis of Fan et al. [3], showed that increased consumption of animal protein is associated with increased risk of T2DM, while substituting red and processed meat protein with plant protein reduced the risk. 

Regarding intervention studies, results from the large school and community-based intervention “The Feel4Diabetes Study”, targeting high risk families for developing T2DM, Manios et al., showed that an approach including counselling sessions for behavior modification was particularly successful by decreasing sugary food items and sedentary time, and increasing breakfast and fruit intake [4]. The study of Amer et al. [5], conducted in adults with prediabetes in Saudi Arabia showed that an 18-month intensive program for lifestyle modification was successful for reversing prediabetes. Interestingly, the study by Rohling et al. [6] highlighted the importance of formula diet and showed that in a 26-week intervention in adults with prediabetes, a combined lifestyle and low carbohydrate/low energy formula diet reduced prediabetes prevalence compared with lifestyle alone. The latest scientific interest regarding the possible effect of fasting diets on glycemic control is addressed by the study of Mohd-Yusof et al [7]. In this eight-week intervention study in adults with T2DM following the Ramadan fasting period, it was shown that a structured nutritional therapy was more effective compared to the standard care in glycemic control. 

Among the observational studies published in the special issue, several interesting findings regarding dietary and lifestyle behaviors in various health ages and special population groups were highlighted. Among the three observational studies in adults, in the prospective study of Bennasar-Veny et al. [8], involving 27,844 adult workers, it was shown that in a five-year monitoring period, lifestyle factors like diet, physical activity, smoking and excessive body weight were associated with persistence of prediabetes and progression to T2DM. In the second cross-sectional study of Hou et al. [9], in apparently healthy adults, a western-type dietary pattern rich in red and processed meat, seafood and fried products was associated with increased risk of prediabetes, while a dietary pattern rich in eggs, milk and coffee was associated with decreased prediabetes risk. The cross-sectional study of Nanri et al. [10] also showed that cooking methods for fish have no influence on the association of fish consumption with glucose metabolism in a large sample of apparently healthy Japanese adults. Regarding adolescents, the cross-sectional study of Kafyra et al. [11], in 766 adolescents from the TEENAGE cohort, a dietary pattern characterized by increased consumption of chicken and sweets and low consumption of fruits was positively associated with elevated insulin levels. Regarding the elderly, in the study of Timm et al. [12], it was highlighted that lifestyle modification for T2DM risk reduction for better glycemic control in older persons might be particularly challenging, as it needs to be more clearly explained compared with people of younger age. Finally, in special population groups, like the Pomaks in Greece, with dietary habits close to those practiced in the Muslim religion, (Farmaki et al. [13]), it was shown that regardless of special dietary choices driven by religion, a dietary pattern rich in sweets, beverages, fruit juices and starchy foods is associated with higher risk of high blood glucose levels and with hypertension. 

Regarding hypertension, three observational studies were published in the special issue. Starting with the baseline data of the PREDIMED study, Gallardo-Alfaro et al. [14], revealed that metabolic syndrome severity was associated with lower levels of physical activity, higher sedentary time and lower adherence to the Mediterranean dietary pattern. Another observational study by Xue et al. [15], in apparently healthy adults, showed that excess visceral body fat is associated with elevated blood pressure, as well as with increased risk of hypertension. Regarding children, the cross-sectional study of Perez-Gimeno et al. [16], in 687 Spanish children and adolescents, revealed that increased consumption of energy dense salty foods was associated with diastolic hypertension independently of any other dietary habit.

In the present special issue, several sociodemographic factors related to adherence and maintenance to a healthier lifestyle pattern in relation to T2DM and hypertension risk reduction are revealed. Results from the large school and community-based intervention “the Feel4Diabetes-study” targeting high-risk families for T2DM, showed that sociodemographic characteristics, certain perceptions, beliefs and attitudes, affected the outcome of the two-year intervention. Specifically, regarding glycemic indices (fasting plasma glucose, insulin and glycosylated hemoglobin levels), Moschonis et al. revealed that those who benefited most were males, younger people, and those with higher education, living in southern and eastern Europe, perceiving their body weight to be higher than normal [17]. Regarding their lipidemic profile, additional results from the Feel4Diabetes-study (Karatzi et al.) showed that women, people living in southeastern Europe, coming from two-parent families, with self-perceived higher financial security, higher educational level, less emotional eating and less sedentary behavior, had a higher probability of benefit from the two-year intervention [18]. Oumrait et al. revealed in a cross-sectional study that there are several social constraints, especially in socio-economically disadvantage populations, that must be addressed by future intervention studies for lifestyle modification [19]. Another important issue highlighted in the study of Tilles-Tirkkonen et al., was eating competence, which reflects food acceptance, positive attitude and general skills related to food and eating. It was shown that eating-competent people present with a higher probability of having better diet quality and lower risk of developing insulin resistance, metabolic syndrome and T2DM [20]. In the observational study of Spires et al., the importance of food environment and especially of food advertisements on food choices were highlighted that may act either enhancing or decreasing the risk of chronic diseases like T2DM and hypertension [21]. 

Another important topic raised in the special issue by three articles was the use of telemedicine and electronic health programs. In this regard the umbrella review by Timpel et al. [22], which gathered data from reviews and metanalyses regarding the use of telemedicine, highlighted the improvements needed in future telemedicine studies to more effectively prevent diabetes and hypertension. Regarding use of e-health programs, two papers by Tragomalou et al., a review and an intervention study, revealed the effectiveness of these programs on both prevention and treatment of cardiometabolic risk factors. Specifically, the intervention study of Tragomalou et al., which used an electronic personalized nutrition program in overweight and obese children and adolescents and their families, showed that after one year of intervention, the study participants significantly reduced their body weight and other cardiometabolic risk factors, such as their glycosylated hemoglobin (HBA1) [23,24].

Finally, an interesting topic highlighted in the special issue is the need for early identification and timely prevention for both T2DM and hypertension. In this regard the study of Kanellakis et al., developed two self-reported tools for early identification of insulin resistance and hypertension [25]. Such tools, which are valid, low cost, noninvasive and easy to apply using simple, self-reported anthropometric indices without biochemical or other clinical measurements, are particularly useful for identifying adults at risk. Such tools could be used in clinical practice and in public health promotion initiatives for screening the population and referring those at risk for further medical assistance.

The present special issue gathers novel scientific findings regarding diet, lifestyle and sociodemographic factors affecting T2DM and hypertension. These findings could support future public health initiatives aiming to deliver holistic and sustainable lifestyle changes for the prevention and management of these chronic health conditions, prioritizing health equity for all.
